# Safety profile and dose-dependent adverse events of upadacitinib in randomized clinical trials: a systematic review and meta-analysis

**DOI:** 10.3389/fphar.2025.1598972

**Published:** 2025-06-23

**Authors:** Cong Zhang, Zhiwen Fu, Jinmei Liu, Shijun Li, Xu Chen, Yi Zhang, Jiyi Xie

**Affiliations:** ^1^ Department of Pharmacy, Union Hospital, Tongji Medical College, Huazhong University of Science and Technology, Wuhan, China; ^2^ Department of Pharmacy, The First People’s Hospital of Jiangxia District, Wuhan, China

**Keywords:** upadacitinib, adverse events, incidence and risk, systematic review, meta-analysis

## Abstract

**Introduction:**

Upadacitinib, one of the Janus kinase (JAK) inhibitors, has gained global approval for treating various inflammatory and autoimmune diseases. However, despite the FDA’s black box warning on all JAK inhibitors, further research is necessary to verify the potential risks associated specifically with upadacitinib. Therefore, this study conducted a comprehensive systematic review and meta-analysis to assess the safety profile of upadacitinib and explore potential dose-related differences.

**Methods:**

Relevant English publications were identified through a comprehensive search of eligible databases conducted in March 2024 and subsequently updated in May 2025. The inclusion criteria focused on randomized controlled trials that included safety data on upadacitinib.

**Results:**

A total of 9,547 patients were involved in this meta-analysis. Upadacitinib treatment was associated with an increased risk of hepatic disorder, neutropenia, acne, herpes zoster, and increased creatine phosphokinase levels. Notably, the risks of hepatic disorder, neutropenia, and acne also exhibited a dose-dependent relationship. However, there was no significant association between upadacitinib treatment and an elevated risk of renal dysfunction, non-melanoma skin cancer (NMSC), major adverse cardiovascular event (MACE), or venous thromboembolic event (VTE).

**Conclusion:**

This study reveals that upadacitinib generally has a favorable safety profile, with increased risks of hepatic disorder, neutropenia, acne, especially at higher doses. There was no significant association with renal dysfunction, NMSC, MACE, or VTE. These findings may serve as an evidence base for potential future modifications or removal of the FDA’s black box warning for upadacitinib.

## Introduction

Janus kinases (JAKs) play a pivotal role in the pathogenesis of autoimmune disorders, including rheumatoid arthritis, ulcerative colitis, psoriatic arthritis, and ankylosing spondylitis (Tektonidou, 2019). The four human JAKs, namely, JAK1, JAK2, JAK3, and TYK2, exhibit distinct cellular distribution patterns, receptor interactions, and biological effects they mediate. These differences contribute to their specificities and significance in the regulation of immune and physiological processes. In recent years, JAK inhibitors have been developed for the treatment of various inflammatory and autoimmune diseases. This class of inhibitors comprises more than a dozen drugs, including tofacitinib, baricitinib, and upadacitinib; however, their safety profile remains a subject of controversy (Szekanecz et al., 2024).

Due to the observed safety concerns, including an elevated risk of serious cardiovascular events and malignancies associated with tofacitinib, the FDA has mandated a black box warning for all JAK inhibitors (Elmariah et al., 2022). However, in contrast to tofacitinib, which primarily targets JAK1 and JAK3, upadacitinib demonstrates a remarkable level of selectivity exclusively towards JAK1. Therefore, the potential augmented risk of severe infections, malignancies, and thrombotic events associated with upadacitinib necessitates further validation through supplementary investigations.

To date, upadacitinib has been approved globally for a number of indications, including: moderately-severe active rheumatoid arthritis, active psoriatic arthritis, moderate-to-severe atopic dermatitis, active ankylosing spondylitis, moderate-to-severe ulcerative colitis, active non-radiographic axial spondyloarthritis, and moderate-to-severe active Crohn’s disease. Several clinical trials investigating upadacitinib for the aforementioned diseases have examined diverse types of adverse events (AEs); however, due to limited patient numbers and varying drug dosages across trials, the incidence and risk associated with different types of upadacitinib-induced AEs remain unknown at present. Therefore, this study undertook a comprehensive systematic review and meta-analysis of safety data pertaining to upadacitinib. We conducted a quantitative synthesis of randomized controlled trials (RCTs) to investigate the incidence and risk of diverse AEs in patients with varying diseases who were treated with upadacitinib. Our objective is to enhance comprehension regarding the safety profile of upadacitinib, provide valuable insights for clinicians, and facilitate rational utilization of this medication.

## Methods

### Literature search

This systematic review and meta-analysis was conducted and reported according to the Preferred Reporting Items for Systematic Reviews and Meta-analyses (PRISMA) guidelines (Page et al., 2021). The protocol was pre-registered at PROSPERO (CRD42024502758). Relevant publications were searched in PubMed, EMBASE, and the Cochrane Library. The systematic review was performed in March 2024 and updated in May 2025.

The keywords for searching included: “upadacitinib”, “Janus kinase inhibitors”, “randomized controlled trial”. References of the selected articles were also checked to identify further eligible trials.

### Study selection criteria

This study excluded non-randomized trials, reviews, editorials, and correspondences. We included only prospective RCTs of upadacitinib in the treatment of atopic dermatitis and autoimmune diseases. Selecting studies that met the inclusion and exclusion criteria was independently performed by two authors, and disagreements were settled by the third author. The inclusion criteria were based on the PICO-framework. In detail, Population (P): atopic dermatitis and autoimmune diseases patients; Intervention (I): treatments by upadacitinib 15 mg or 30 mg; Comparison (C): placebo. Outcomes (O): any AEs.

### Outcomes

Safety outcomes of all included studies, including serious AE (any unfavourable medical occurrence that is considered serious at any dose if it results in death/is life-threatening/requires inpatient hospitalisation or prolongation of existing hospitalization/results in persistent or significant disability/incapacity/is a congenital anomaly/birth defect), AE leading to discontinuation, opportunistic infection, herpes zoster, hepatic disorder, renal dysfunction, increased creatine phosphokinase (CPK), headache, nausea, anaemia, neutropenia, lymphopenia, acne, non-melanoma skin cancer (NMSC), malignancy other than NMSC, atopic dermatitis, major adverse cardiovascular event (MACE), venous thromboembolic event (VTE) and deaths.

This study compares the safety outcomes of two different doses of upadacitinib (15 mg and 30 mg) with placebo, respectively, in addition to the difference between upadacitinib 15 mg and 30 mg.

### Quality assessment

The risk of bias in each included RCTs was assessed independently by two authors (C. Zhang, J.Y. Xie) using the revised Cochrane Risk of Bias tool (version 2.0). Disagreements were discussed and resolved by consensus between both reviewers or via consultation with a third reviewer (Z.W. Fu).

### Statistical analysis

Results were quantitatively synthesized by means of meta-analysis using the Review Manager (version 5.4; Cochrane Collaboration, Oxford, England). The Mantel-Haenszel method was used to estimate the pooled risk ratio (RR) for each safety outcomes. I^2^ was used to evaluate heterogeneity across studies. When heterogeneity (I^2^ ≥ 50%) was detected, random-effects meta-analyses were performed. I^2^ < 50%, a fixed-effect statistical model was used. Results obtained from the analyses were displayed by generating a forest plot. A p-value of < 0.05 was considered statistically significant.

## Results

### Study selection and trial characteristics

By conducting a comprehensive review of the literature, we amassed 1,505 records pertaining to upadacitinib across mentioned databases. After eliminating 741 duplicates, we further sifted through the remaining records, discarding basic researches (n = 450), letters (n = 162), correspondences (n = 54), and others (n = 63). This rigorous filtering process left us with a final tally of 35 pertinent records.

**FIGURE 1:**
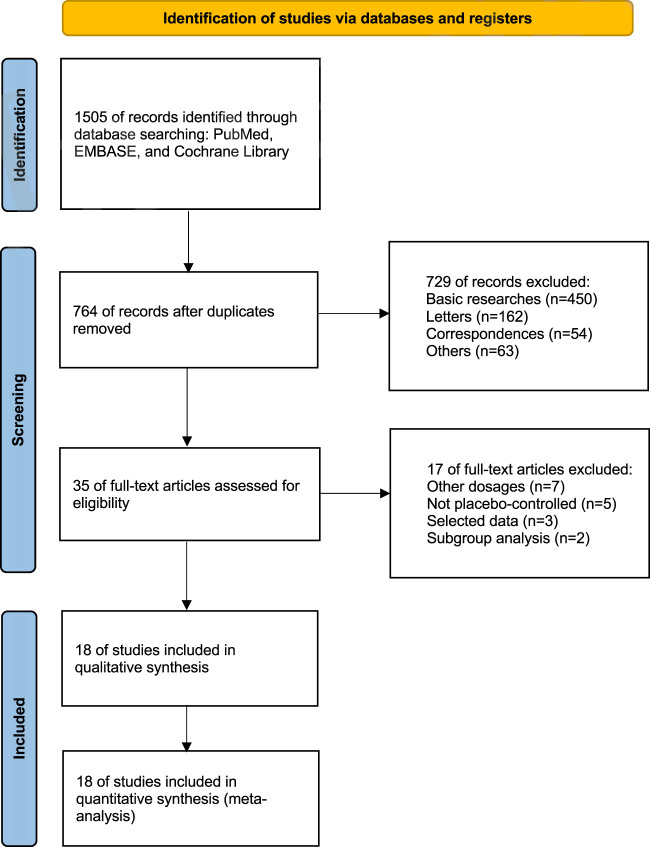
The PRISMA flow diagram of the safety analysis.

Upon thorough evaluation of the full-text articles, 17 records were ruled out for failing to satisfy the inclusion criteria, including records with other dosages (n = 7), not placebo-controlled (n = 5), selected data (n = 3), and subgroup analysis (n = 2) (Figure 1).

Ultimately, we included 18 eligible studies for quantitative analysis (Table 1). 11 studies involved both 15 mg and 30 mg doses, six studies involved only the 15 mg dose, and one study involved only the 30 mg dose. A total of 9,547 patients were involved in this meta-analysis, of which 5,907 patients received upadacitinib (15 mg:3,658 patients; 30 mg:2,249 patients), while the remaining 3,640 patients received placebo. According to the type of disease, including ankylosing spondylitis (607 patients), atopic dermatitis (2,708 patients), psoriatic arthritis (1,916 patients), non-radiographic axial spondyloarthritis (313 patients), rheumatoid arthritis (2,947 patients), ulcerative colitis (598 patients), giant-cell arteritis (321 patients) and systemic lupus erythematosus (137 patients).

**TABLE 1:** Characteristics of included studies (n = 18)*.

Author	Year	NCT NO.	Study	Phase	Disease	Treatment	Dose (mg)	Total patients	Control	Total patients	Follow-up (week)
van der Heijde et al. (2019)	2019	NCT03178487	SELECT-AXIS 1	3	AS	Upadacitinib	15	93	Placebo	94	14
Guttman-Yassky et al. (2021)	2021	NCT03569293	Measure Up 1	3	AD	Upadacitinib	15/30	281/285	Placebo	281	16
Guttman-Yassky et al. (2021)	2021	NCT03607422	Measure Up 2	3	AD	Upadacitinib	15/30	276/282	Placebo	278	16
Reich et al. (2021)	2021	NCT03568318	AD Up	3	AD	Upadacitinib	15/30	300/297	Placebo	303	16
Guttman-Yassky et al. (2020)	2020	NCT02925117	NA	2b	AD	Upadacitinib	15/30	42/42	Placebo	41	16
Mease et al. (2021)	2020	NCT03104374	SELECT-PsA 2	3	PsA	Upadacitinib	15/30	211/218	Placebo	212	24
McInnes et al. (2021)	2021	NCT03104400	SELECT-PsA 1	3	PsA	Upadacitinib	15/30	429/423	Placebo	423	24
Deodhar et al. (2022)	2022	NCT04169373	SELECT-AXIS 2	3	NR-axSpA	Upadacitinib	15	156	Placebo	157	14
van der Heijde et al. (2022a)	2022	NCT04169373	AS bDMARD-IR	3	AS	Upadacitinib	15	211	Placebo	209	14
Zeng et al. (2021)	2021	NCT02955212	NA	3	RA	Upadacitinib	15	169	Placebo	169	12
Sandborn et al. (2020)	2020	NCT02819635	U-ACHIEVE substudy 1	2b	UC	Upadacitinib	15/30	49/52	Placebo	46	8
Genovese et al. (2018)	2018	NCT02706847	SELECT-BEYOND	3	RA	Upadacitinib	15/30	164/165	Placebo	169	24
Burmester et al. (2018)	2018	NCT02675426	SELECT-NEXT	3	RA	Upadacitinib	15/30	221/219	Placebo	221	12
Fleischmann et al. (2019)	2019	NCT02629159	SELECT-COMPARE	3	RA	Upadacitinib	15	650	Placebo	652	26
Kameda et al. (2020)	2020	NCT02720523	SELECT-SUNRISE	2b/3	RA	Upadacitinib	15/30	49/50	Placebo	49	12
Danese et al. (2022)	2022	NCT02819635	U-ACHIEVE substudy 3	3	UC	Upadacitinib	15/30	148/154	Placebo	149	52
Blockmans et al. (2025)	2025	NCT03725202	SELECT-GCA	3	GCA	Upadacitinib	15	209	Placebo	112	52
Merrill et al. (2024)	2024	NCT03978520	SLEek	2	SLE	Upadacitinib	30	62	Placebo	75	48

* Measure Up 1 (NCT03569293) and Measure Up 2 (NCT03607422) were reported in a single publication.

NCT: national clinical trial, NA: not applicable, AS: ankylosing spondylitis, AD: atopic dermatitis, PsA: psoriatic arthritis, NR-axSpA: non-radiographic axial spondyloarthritis, RA: rheumatoid arthritis, UC: ulcerative colitis, GCA: Giant-cell Arteritis, SLE: systemic lupus erythematosus.

### Risk of bias and quality assessment

**FIGURE 2:**
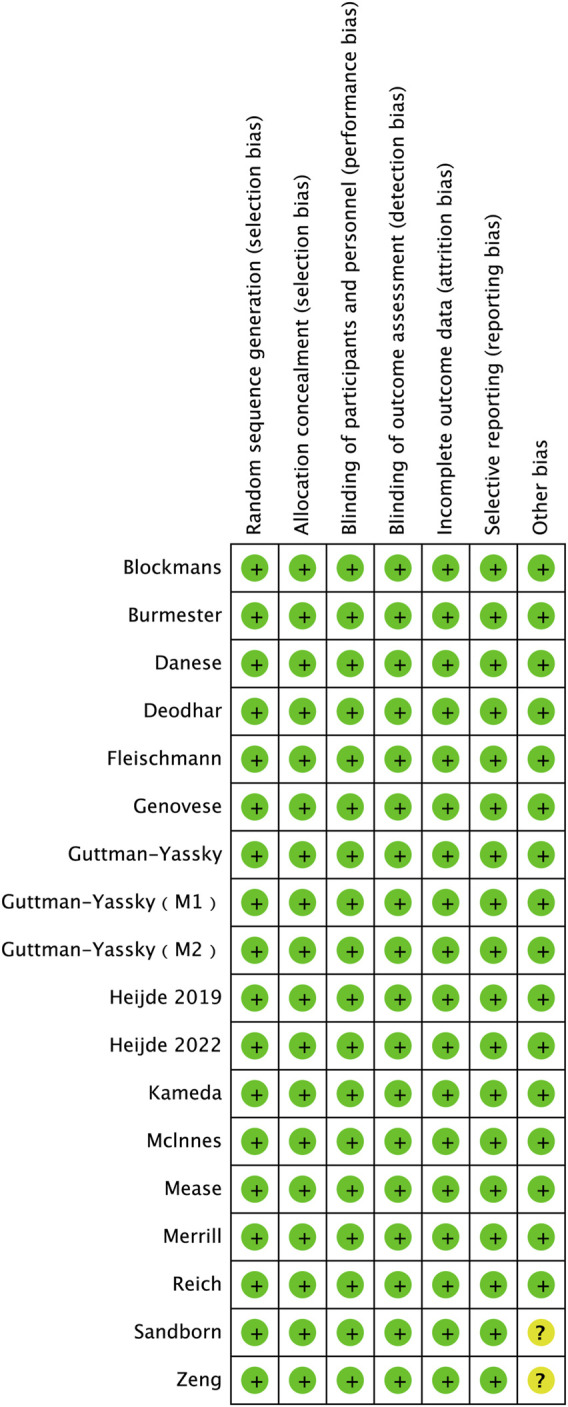
Risk of bias summary.

The revised Cochrane Risk of Bias tool (RoB version 2.0) was utilized to evaluate the quality of each study included in our analysis. The outcomes of the quality assessment revealed that all of the incorporated studies presented a low risk of bias (Figure 2).

### Safety profile of upadacitinib 15 mg versus placebo

A total of 23 AE-related entries were included in this safety analysis (Table 2). Of these, there was a statistically significant difference in the incidence of any AE, herpes zoster, hepatic disorder, increased CPK, neutropenia, acne, and atopic dermatitis, upadacitinib 15 mg versus placebo. With the exception of atopic dermatitis, treatment with the 15 mg dose of upadacitinib resulted in a higher incidence of these AEs than placebo.

**TABLE 2:** Safety profile of upadacitinib 15 mg versus placebo.

Adverse events	Number of trials	Upadacitinib (15 mg)			Placebo			Heterogeneity analysis			Statistical analysis model	Statistical analysis	
		Events	Total	Incidence	Events	Total	Incidence	Chi^2^	P	I^2^		RR (95%CI)	P
Any adverse event	17	2325	3658	63.56%	2027	3564	56.87%	22.95	0.12	30%	Fixed	1.10 (1.06, 1.14)	<0.00001^*^
Serious adverse event	16	157	3447	4.55%	120	3352	3.58%	17.97	0.26	17%	Fixed	1.12 (0.89, 1.41)	0.33
Adverse event leading to discontinuation	17	139	3609	3.85%	147	3515	4.18%	17.10	0.31	12%	Fixed	0.84 (0.67, 1.06)	0.14
Any infection	11	714	2275	31.38%	589	2272	25.92%	19.82	0.03	50%	Random	1.16 (1.00, 1.34)	0.05
Serious infection	16	57	3565	1.60%	38	3470	1.10%	13.13	0.59	0%	Fixed	1.26 (0.86, 1.86)	0.23
Opportunistic infection^a^	12	20	2989	0.67%	11	2897	0.38%	8.74	0.65	0%	Fixed	1.46 (0.78, 2.74)	0.24
Nasopharyngitis	7	114	1361	8.38%	92	1366	6.73%	1.32	0.97	0%	Fixed	0.02 (−0.00, 0.04)	0.10
Upper respiratory tract infection	7	89	1361	6.54%	76	1366	5.56%	3.25	0.78	0%	Fixed	1.17 (0.87, 1.57)	0.29
Herpes zoster	14	52	3474	1.50%	21	3384	0.62%	6.72	0.92	0%	Fixed	2.12 (1.31, 3.46)	0.002^*^
Hepatic disorder	17	161	3658	4.40%	102	3564	2.86%	11.06	0.81	0%	Fixed	1.52 (1.19, 1.93)	0.0007^*^
Renal dysfunction	5	6	1297	0.46%	6	1199	0.50%	1.28	0.86	0%	Fixed	0.75 (0.27, 2.07)	0.58
Increased creatine phosphokinase	12	117	2428	4.82%	37	2328	1.59%	9.78	0.55	0%	Fixed	3.01 (2.10, 4.31)	<0.00001^*^
Headache	7	68	1361	4.99%	59	1366	4.32%	4.33	0.63	0%	Fixed	1.16 (0.82, 1.63)	0.40
Nausea	3	18	356	5.06%	13	355	3.66%	4.49	0.11	55%	Random	0.95 (0.20, 4.60)	0.95
Anaemia	12	44	2660	1.65%	33	2560	1.29%	6.48	0.84	0%	Fixed	1.16 (0.74, 1.81)	0.52
Neutropenia	13	41	2746	1.49%	11	2648	0.41%	5.55	0.94	0%	Fixed	3.03 (1.68, 5.46)	0.0002^*^
Lymphopenia	10	19	2221	0.85%	15	2119	0.71%	3.93	0.92	0%	Fixed	1.16 (0.61, 2.21)	0.66
Acne	5	90	1047	8.60%	25	1051	2.38%	9.23	0.06	57%	Random	3.01 (1.44, 6.31)	0.003^*^
NMSC	7	9	1773	0.51%	5	1672	0.30%	3.38	0.76	0%	Fixed	1.22 (0.49, 3.01)	0.67
Malignancy other than NMSC	6	10	1330	0.75%	3	1234	0.24%	1.29	0.94	0%	Fixed	1.93 (0.70, 5.34)	0.20
Atopic dermatitis	4	30	899	3.34%	74	902	8.20%	2.14	0.54	0%	Fixed	0.41 (0.27, 0.62)	<0.0001^*^
MACE	6	2	1811	0.11%	7	1717	0.41%	4.26	0.51	0%	Fixed	0.45 (0.16, 1.28)	0.14
VTE	6	11	1944	0.56%	7	1846	0.38%	2.14	0.83	0%	Fixed	1.07 (0.46, 2.48)	0.88

^a^ excluding tuberculosis and herpes zoster, *: p < 0.05.

NMSC: non-melanoma skin cancer, MACE: major adverse cardiovascular events (defined as non-fatal myocardial infarction, non-fatal stroke and cardiovascular death), VTE: venous thromboembolic event (defined as deep vein thrombosis and pulmonary embolism).

### Safety profile of upadacitinib 30 mg versus placebo

A total of 23 AE-related entries were included in this safety analysis (Table 3). Of these, there was a statistically significant difference in the incidence of any AE, any infection, serious infection, nasopharyngitis, upper respiratory tract infection, herpes zoster, hepatic disorder, increased CPK, neutropenia, lymphopenia, acne, malignancy other than NMSC and atopic dermatitis, upadacitinib 30 mg versus placebo. With the exception of atopic dermatitis, treatment with the 30 mg dose of upadacitinib resulted in a higher incidence of these AEs than placebo.

**TABLE 3:** Safety profile of upadacitinib 30 mg versus placebo.

Adverse events	Number of trials	Upadacitinib (30 mg)			Placebo			Heterogeneity analysis			Statistical analysis model	Statistical analysis	
		Events	Total	Incidence	Events	Total	Incidence	Chi^2^	P	I^2^		RR (95%CI)	P
Any adverse event	12	1625	2314	70.22%	1350	2246	60.11%	13.11	0.29	16%	Fixed	1.17 (1.12, 1.22)	<0.00001^*^
Serious adverse event	11	95	2096	4.53%	81	2034	3.98%	21.91	0.02	54%	Random	0.99 (0.60, 1.63)	0.96
Adverse event leading to discontinuation	12	123	2314	5.31%	101	2246	4.50%	16.19	0.13	32%	Fixed	1.18 (0.91, 1.52)	0.21
Any infection	7	478	1234	38.74%	346	1160	29.83%	14.05	0.03	57%	Random	1.30 (1.07, 1.59)	0.009^*^
Serious infection	11	43	2272	1.89%	22	2206	1.00%	13.91	0.18	28%	Fixed	1.83 (1.13, 2.95)	0.01^*^
Opportunistic infection^a^	9	19	1836	1.03%	7	1779	0.39%	7.24	0.51	0%	Fixed	2.07 (1.01, 4.26)	0.05
Nasopharyngitis	6	129	1279	10.09%	88	1272	6.92%	2.48	0.78	0%	Fixed	1.46 (1.13, 1.89)	0.004^*^
Upper respiratory tract infection	6	104	1279	8.13%	73	1272	5.74%	2.20	0.82	0%	Fixed	1.42 (1.06, 1.89)	0.02^*^
Herpes zoster	10	46	2155	2.13%	16	2160	0.74%	4.55	0.87	0%	Fixed	2.82 (1.62, 4.90)	0.0002^*^
Hepatic disorder	12	104	2314	4.49%	45	2246	2.00%	16.83	0.11	35%	Fixed	2.24 (1.60, 3.14)	<0.00001^*^
Renal dysfunction	4	3	857	0.35%	3	859	0.35%	1.08	0.78	0%	Fixed	1.03 (0.26, 4.04)	0.96
Increased creatine phosphokinase	9	126	2037	6.19%	34	1953	1.74%	5.92	0.66	0%	Fixed	3.52 (2.43, 5.09)	<0.00001^*^
Headache	6	69	1279	5.39%	57	1272	4.48%	6.04	0.30	17%	Fixed	1.20 (0.85, 1.70)	0.29
Anaemia	10	60	2099	2.86%	28	2028	1.38%	18.23	0.03	51%	Random	1.74 (0.83, 3.64)	0.14
Neutropenia	9	72	1982	3.63%	9	1982	0.45%	2.51	0.96	0%	Fixed	7.30 (3.78, 14.11)	<0.00001^*^
Lymphopenia	8	30	1940	1.55%	11	1942	0.57%	3.79	0.80	0%	Fixed	2.47 (1.31, 4.69)	0.005^*^
Acne	5	143	1060	13.49%	25	1051	2.38%	10.84	0.03	63%	Random	4.79 (2.25, 10.17)	<0.0001^*^
NMSC	5	5	1311	0.38%	1	1308	0.08%	0.07	1.00	0%	Fixed	2.60 (0.61, 11.16)	0.20
Malignancy other than NMSC	8	12	2043	0.59%	1	2036	0.05%	0.42	1.00	0%	Fixed	3.42 (1.20, 9.75)	0.02^*^
Atopic dermatitis	4	14	906	1.55%	74	902	8.20%	8.67	0.03	65%	Random	0.23 (0.08, 0.68)	0.007^*^
Deaths	3	1	806	0.12%	2	804	0.25%	1.25	0.53	0%	Fixed	0.71 (0.14, 3.59)	0.68
MACE	4	2	858	0.23%	3	868	0.34%	1.35	0.72	0%	Fixed	0.81 (0.20, 3.24)	0.77
VTE	3	3	859	0.35%	2	850	0.24%	1.47	0.48	0%	Fixed	1.31 (0.29, 5.86)	0.72

^a^ excluding tuberculosis and herpes zoster, *: p < 0.05.

NMSC: non-melanoma skin cancer, MACE: major adverse cardiovascular events (defined as non-fatal myocardial infarction, non-fatal stroke and cardiovascular death), VTE: venous thromboembolic event (defined as deep vein thrombosis and pulmonary embolism).

### Safety profile of upadacitinib 30 mg versus upadacitinib 15 mg

**FIGURE 3:**
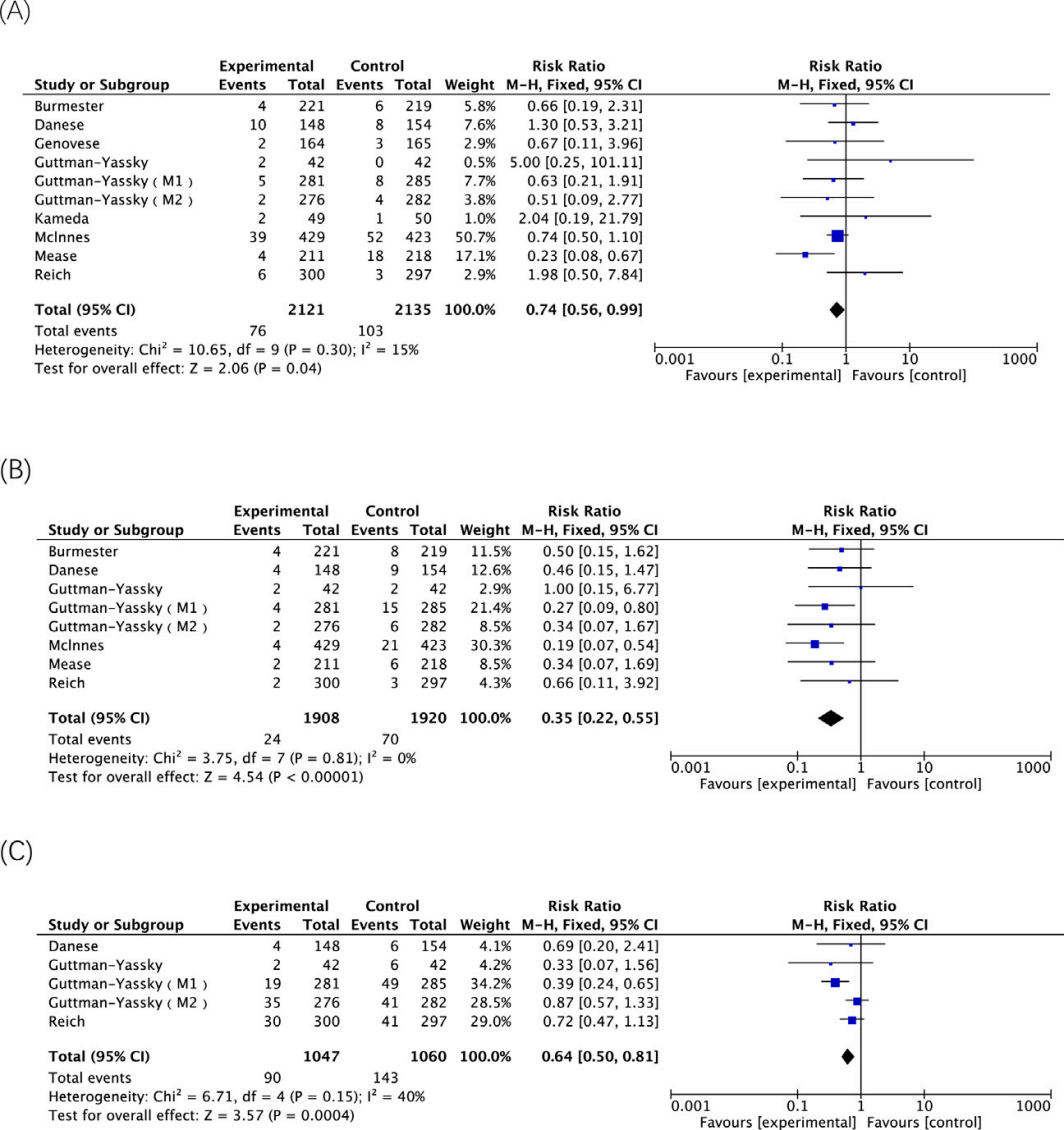
The meta-analysis of the incidence of dose-dependent AEs related to upadacitinib in eligible RCTs. **(A)** Incidences of hepatic disorder caused by upadacitinib 15 mg versus 30 mg; **(B)** Incidences of neutropenia caused by upadacitinib 15 mg versus 30 mg; **(C)** Incidences of acne caused by upadacitinib 15 mg versus 30 mg.

A total of 22 AE-related entries were included in this safety analysis (Table 4). Of these, there was a statistically significant difference in the incidence of any AE, AE leading to discontinuation, any infection, hepatic disorder, neutropenia, lymphopenia, acne and atopic dermatitis, upadacitinib 30 mg versus 15 mg. With the exception of atopic dermatitis, treatment with the 30 mg dose of upadacitinib resulted in a higher incidence of these AEs than the 15 mg dose. Among them, the two doses respectively have risks of occurrence and dose-dependent AEs, including hepatic disorder, neutropenia and acne (Figure 3).

**TABLE 4:** Safety profile of upadacitinib 15 mg versus 30 mg.

Adverse events	Number of trials	Upadacitinib (15 mg)			Upadacitinib (30 mg)			Heterogeneity analysis			Statistical analysis model	Statistical analysis	
		Events	Total	Incidence	Events	Total	Incidence	Chi^2^	P	I^2^		RR (95%CI)	P
Any adverse event	11	1385	2170	63.82%	1574	2252	69.89%	12.64	0.24	21%	Fixed	0.91 (0.88, 0.95)	<0.0001^*^
Serious adverse event	10	49	1959	2.50%	56	2032	2.76%	5.38	0.72	0%	Fixed	0.93 (0.64, 1.36)	0.72
Adverse event leading to discontinuation	11	69	2170	3.18%	117	2252	5.20%	10.40	0.41	4%	Fixed	0.61 (0.46, 0.82)	0.0010^*^
Any infection	7	402	1165	34.51%	478	1234	38.74%	6.59	0.36	9%	Fixed	0.87 (0.78, 0.96)	0.009^*^
Serious infection	11	22	2170	1.01%	36	2252	1.60%	8.96	0.54	0%	Fixed	0.65 (0.39, 1.08)	0.09
Opportunistic infection^a^	9	9	2079	0.43%	18	2093	0.86%	6.60	0.58	0%	Fixed	0.58 (0.28, 1.17)	0.13
Nasopharyngitis	6	109	1268	8.60%	129	1279	10.09%	1.34	0.93	0%	Fixed	0.85 (0.67, 1.09)	0.20
Upper respiratory tract infection	6	89	1268	7.02%	104	1279	8.13%	2.09	0.84	0%	Fixed	0.86 (0.66, 1.13)	0.29
Herpes zoster	9	29	2079	1.39%	42	2093	2.01%	5.79	0.67	0%	Fixed	0.70 (0.44, 1.12)	0.14
Hepatic disorder	10	76	2121	3.58%	103	2135	4.82%	10.65	0.30	15%	Fixed	0.74 (0.56, 0.99)	0.04^*^
Renal dysfunction	3	2	659	0.30%	2	669	0.30%	0.87	0.65	0%	Fixed	1.02 (0.21, 5.07)	0.98
Increased creatine phosphokinase	9	100	1957	5.11%	126	2037	6.19%	4.35	0.82	0%	Fixed	0.82 (0.63, 1.05)	0.12
Headache	6	63	1268	4.97%	69	1279	5.39%	1.06	0.96	0%	Fixed	0.92 (0.66, 1.28)	0.63
Anaemia	9	21	1957	1.07%	58	2037	2.85%	16.88	0.03	53%	Random	0.45 (0.20, 1.04)	0.06
Neutropenia	8	24	1908	1.26%	70	1920	3.65%	3.75	0.81	0%	Fixed	0.35 (0.22, 0.55)	<0.00001^*^
Lymphopenia	7	14	1608	0.87%	28	1623	1.73%	3.57	0.73	0%	Fixed	0.52 (0.28, 0.96)	0.04^*^
Acne	5	90	1047	8.60%	143	1060	13.49%	6.71	0.15	40%	Fixed	0.64 (0.50, 0.81)	0.0004^*^
NMSC	7	4	1866	0.21%	7	1478	0.47%	10.40	0.11	42%	Fixed	0.54 (0.24, 1.25)	0.15
Malignancy other than NMSC	8	5	2030	0.25%	12	2043	0.59%	1.28	0.99	0%	Fixed	0.51 (0.21, 1.25)	0.14
Atopic dermatitis	4	30	899	3.34%	14	906	1.55%	4.53	0.21	34%	Fixed	2.16 (1.15, 4.04)	0.02^*^
MACE	3	2	596	0.34%	1	602	0.17%	1.24	0.54	0%	Fixed	1.41 (0.28, 7.10)	0.68
VTE	3	1	788	0.13%	3	795	0.38%	1.62	0.45	0%	Fixed	0.57 (0.12, 2.65)	0.47

^a^ excluding tuberculosis and herpes zoster, *: p < 0.05.

NMSC: non-melanoma skin cancer, MACE: major adverse cardiovascular events (defined as non-fatal myocardial infarction, non-fatal stroke and cardiovascular death), VTE: venous thromboembolic event (defined as deep vein thrombosis and pulmonary embolism).

### Safety profile of subgroup analysis based on diseases

Based on the above results, the results with greater heterogeneity were selected for subgroup analysis according to disease types. The heterogeneity decreased in all cases, and the vast majority decreased to 0%. A fixed-effect model was adopted. The results indicated that both 15 mg and 30 mg doses of upadacitinib might increase the risk of infection in RA patients (p < 0.00001, p = 0.005). The risk of acne may increase in AD patients (p < 0.00001). In addition, a 30 mg dose of upadacitinib may increase the risk of anaemia in AD patients (p = 0.04), while there is no statistically significant difference in the risk of severe infection compared with placebo (p = 0.29) (Table 5).

**TABLE 5:** Safety profile of subgroup analysis based on diseases.

Adverse events	Number of trials	Disease	Experimental	Control	Heterogeneity analysis			Statistical analysis model	Statistical analysis	
					Chi^2^	P	I^2^		RR (95%CI)	P
Any infection	4	RA	Upadacitinib 15 mg	Placebo	2.67	0.44	0%	Fixed	1.38 (1.20, 1.58)	<0.00001^*^
	3	RA	Upadacitinib 30 mg	Placebo	3.34	0.19	40%	Fixed	1.36 (1.10, 1.67)	0.005^*^
Acne	4	AD	Upadacitinib 15 mg	Placebo	1.54	0.67	0%	Fixed	4.54 (2.79, 7.40)	<0.00001^*^
	4	AD	Upadacitinib 30 mg	Placebo	0.15	0.99	0%	Fixed	7.17 (4.48, 11.49)	<0.00001^*^
Anaemia	4	AD	Upadacitinib 30 mg	Placebo	0.44	0.93	0%	Fixed	2.97 (1.03, 8.59)	0.04^*^
	4	AD	Upadacitinib 15 mg	Upadacitinib 30 mg	0.77	0.86	0%	Fixed	0.29 (0.10, 0.87)	0.03^*^
Serious adverse event	4	AD	Upadacitinib 30 mg	Placebo	1.38	0.71	0%	Fixed	0.73 (0.41, 1.30)	0.29

*: p < 0.05.

RA: rheumatoid arthritis, AD: atopic dermatitis.

### Adverse events highlighted in black box warnings

**FIGURE 4:**
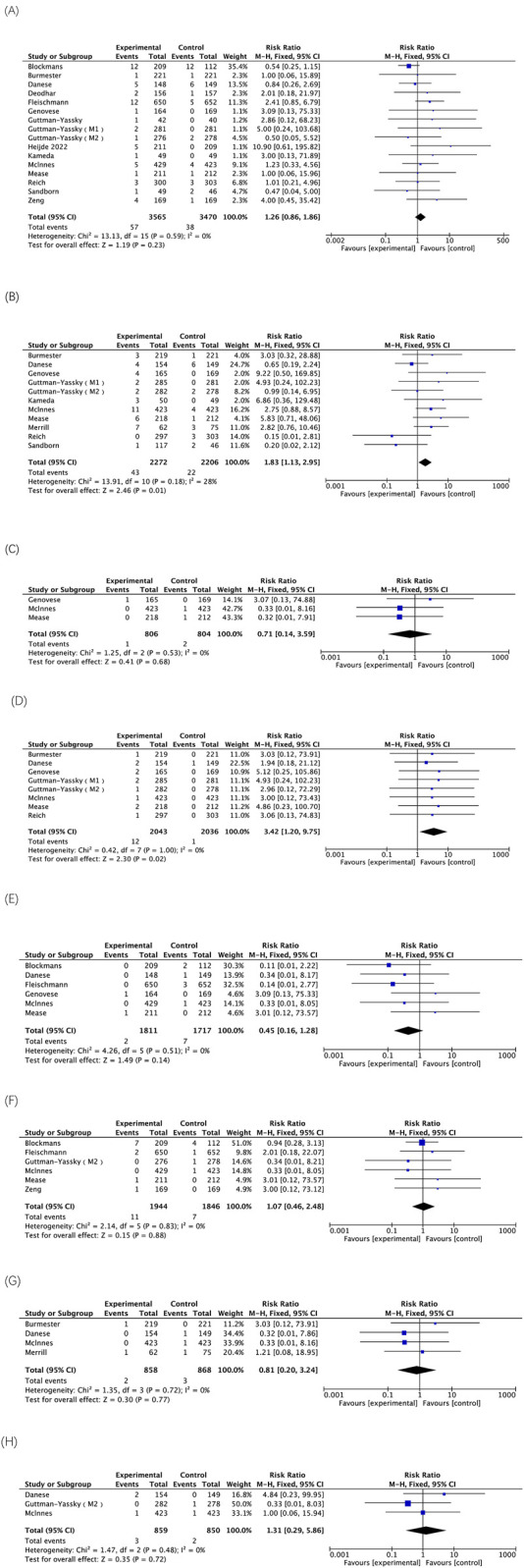
The meta-analysis of the incidence of AEs highlighted in black box warnings associated with upadacitinib in eligible RCTs. **(A)** Incidences of serious infection caused by upadacitinib 15 mg versus placebo; **(B)** Incidences of serious infection caused by upadacitinib 30 mg versus placebo; **(C)** Incidences of deaths caused by upadacitinib 30 mg versus placebo; **(D)** Incidences of malignancy other than NMSC caused by upadacitinib 30 mg versus placebo; **(E)** Incidences of MACE caused by upadacitinib 15 mg versus placebo; **(F)** Incidences of VTE caused by upadacitinib 15 mg versus placebo; **(G)** Incidences of MACE caused by upadacitinib 30 mg versus placebo; **(H)** Incidences of VTE caused by upadacitinib 30 mg versus placebo.

The five AEs highlighted in the black box warning for upadacitinib include serious infection, death, malignancy, MACE, and thrombosis. The analysis of the included RCTs involving treatment with either 15 mg or 30 mg of upadacitinib demonstrated that the use of 30 mg of upadacitinib may elevate the risk of serious infections. Three RCTs involving treatment with 30 mg of upadacitinib were included, the treatment group comprised 806 participants, with 1 death reported. There was no statistically significant difference between the treatment and placebo groups. The results of 8 RCTs indicate that the 30 mg dose of upadacitinib may be associated with an increased risk of malignancy other than NMSC. However, the 15 mg dose of upadacitinib does not show statistically significant associations with such risks. Treatment with both doses of upadacitinib did not result in a statistically significant difference in the risk of MACE or VTE (Figure 4).

## Discussion

Based on the results of 18 clinical studies involving 9,547 patients, the overall incidence of any AE was observed to be 63.56% or 70.22% in patients treated with upadacitinib at doses of 15 mg or 30 mg, respectively. These rates were found to be higher compared to the placebo group, while the incidence of serious AEs was reported as 4.55% or 4.53%, respectively, which exhibited a slightly elevated but statistically insignificant difference when compared to the placebo group (p = 0.33; p = 0.96). The results of this study indicate that treatment with either the 15 mg or 30 mg doses of upadacitinib exhibits a favorable safety profile overall. The 2-year long-term safety and efficacy study (van der Heijde et al., 2022b) demonstrated a favorable benefit-risk profile of upadacitinib, which is consistent with the overall safety findings observed in this study.

As the dosage is doubled, treatment with upadacitinib 30 mg demonstrates an elevated risk of AE leading to discontinuation (RR = 0.61; 95%CI, 0.46–0.82; p = 0.0010) and infection (RR = 0.87; 95%CI, 0.78–0.96; p = 0.009) compared to treatment with 15 mg. However, no statistically significant differences were observed between the two doses in terms of serious infection, opportunistic infection, nasopharyngitis and upper respiratory tract infection. A systematic literature review that included different types of studies, such as RCTs, safety trials, *post hoc* analyses, conference abstracts, and real-world cohort studies, indicated that the use of upadacitinib could observe serious infection events, but the dose-dependence was not significant (Konzett et al., 2025). Similarly, a comprehensive analytical study (Guttman-Yassky et al., 2023) on the safety of upadacitinib in patients with moderate-to-severe atopic dermatitis demonstrated that during an average treatment duration of approximately 1 year, the likelihood of AE leading to discontinuation was higher in the 30 mg group compared to the 15 mg group. Moreover, both dosages exhibited comparable risks for inducing severe infection. Although both upadacitinib and TNF-α inhibitors may increase the risk of opportunistic infections through immunosuppression, their specific molecular targets and signaling pathways differ (Murdaca et al., 2019). Regarding the elevated risk of opportunistic infections, findings from this study demonstrating results for the 30 mg dose of upadacitinib versus placebo (RR = 2.07; 95% CI, 1.01–4.26; p = 0.05) merit significant attention.

The findings also indicate that treatment with either upadacitinib 15 mg or 30 mg may be associated with an increased risk of herpes zoster development, and this AE does not appear to be dose-dependent, as there was no statistically significant difference in the incidence of herpes zoster between the two doses (RR = 0.70; 95% CI, 0.44–1.12; p = 0.14). The results of a meta-analysis of the infection risk related to JAK inhibitors in rheumatoid arthritis indicated that the infection risk of JAK inhibitors seemed similar (Alves et al., 2022). Most JAK inhibitors, including upadacitinib, increased the risk of herpes zoster, while this article focused on the broader types of adverse reaction risks of upadacitinib. Moreover, more disease types were included, and the conclusion also confirmed that upadacitinib increases the risk of herpes zoster. Pooled analyses of six phase III clinical studies revealed a higher incidence of herpes zoster in the upadacitinib-treated group compared to those treated with methotrexate monotherapy or adalimumab-combined methotrexate, and within the upadacitinib group, a higher incidence was observed in the 30 mg dose group than in the 15 mg dose group (Winthrop et al., 2022). Therefore, further validation is warranted to ascertain whether the escalated dosage of upadacitinib entails an elevated risk of herpes zoster development. The correlation between different underlying diseases, diverse populations, and the incidence of herpes zoster necessitates further comprehensive analysis. There was once a case report from Japan. A patient who took upadacitinib orally for rheumatoid arthritis for 1 month developed papules and nodules on the head and was diagnosed with moluminatum contagiosa. Three months after discontinuing upadacitinib, the skin lesions improved (Kawano et al., 2022). The reason may be related to upadacitinib’s inhibition of the JAK-STAT (signal transducer and activator of transcription) pathway (i.e., the type I interferon transduction pathway), thereby reducing the antiviral effect and leading to the occurrence of moluminatum contagiosa. A real-world study has identified ADE signals not mentioned in the drug instructions of upadacitinib, such as urogenital system and breast diseases, and the most common signal of serious adverse reactions is urinary tract infection (Wu et al., 2023). These findings require further investigation into their causal relationships. In conclusion, in addition to herpes zoster, other clinically related infection risks should also be noted in immunosuppressed patients using upadacitinib.

Treatment with either upadacitinib 15 mg or 30 mg may be associated with an increased risk of hepatic disorder, and this AE is also dose-dependent, as the incidence of hepatic disorder rises proportionally to the doubling of the dosage (RR = 0.74; 95% CI, 0.56–0.99; p = 0.04). Conversely, the use of upadacitinib has a minimal impact on renal function, as evidenced by no significant difference in the occurrence of renal dysfunction observed between placebo controls for each dosage or between the two dosage groups. As stated in the upadacitinib instructions, no dosage adjustment is necessary for patients with mild, moderate, or severe renal impairment in rheumatoid arthritis, psoriatic arthritis, ankylosing spondylitis, and non-radiographic axial spondyloarthritis. Additionally, no dose adjustment is required for patients with mild-to-moderate renal impairment in any other indication. The pharmacokinetic study of upadacitinib in subjects with renal insufficiency revealed no safety concerns, thereby establishing its suitability for clinical use (Mohamed et al., 2019).

In the controlled study with placebo, upadacitinib demonstrated an association with increased CPK levels, resulting in a 5% incidence when treated with 15 mg compared to a 6.19% incidence when treated with a 30 mg dose. However, there was no significant correlation between the incidence of increased CPK and the dosage of upadacitinib (RR = 0.82; 95% CI, 0.63–1.05; p = 0.12). It is worth noting that a case report has documented the development of myopathy symptoms and elevated blood CPK levels in a patient with Crohn’s disease following an adjustment in daily upadacitinib dosage from 30 mg to 45 mg for 1 week. However, discontinuation of this dose and subsequent initiation of treatment with upadacitinib at 15 mg successfully restored normal blood CPK levels (Schuitema et al., 2024). The precise mechanism underlying the potential elevation of CPK by upadacitinib remains elusive; however, it is postulated to be associated with the drug’s immunomodulatory effects or its ability to induce specific types of muscle damage or inflammation. Nevertheless, the exact etiology of this adverse effect may involve multiple factors, including direct impact on muscle tissue and indirect modulation of inflammation or immune response.

Based on the findings of this study, both 15 mg and 30 mg doses of upadacitinib exhibited a correlation with neutropenia, while the administration of 30 mg doses was associated with an elevated risk of lymphocytopenia. These two adverse reactions demonstrated a dose-dependent relationship. In a phase III clinical trial involving patients with Crohn’s disease, the incidence of neutropenia was higher in the 30 mg upadacitinib group compared to other maintenance groups (Loftus et al., 2023). Similarly, in another study involving patients with ulcerative colitis, the 30 mg upadacitinib group exhibited an elevated risk of neutropenia (Vermeire et al., 2023).

On the skin system, upadacitinib administration is associated with an elevated risk of acne, and this risk escalates proportionally with increasing dosage (RR = 0.64; 95% CI, 0.50–0.81; p = 0.0004). However, the results show certain heterogeneity, which may be related to the inclusion of different disease populations. Therefore, in this study, subgroup analysis was conducted based on AD patients, and four studies were included, I^2^ = 0%. It is suspected that the reason for the heterogeneity is the study on UC patients (Danese et al., 2022). The results of this study are similar to the conclusion of an observational study indicating that upadacitinib treatment increases acne incidence in AD patients, but not in those with joint or gastrointestinal inflammatory diseases (Thyssen et al., 2022). Mechanism of action is related to the possibility that immunosuppression by JAK1 inhibitors may increase or alter microbial colonization of the skin. In the context of *post hoc* pooled analyses from three phase 3 RCTs, the incidence rates of acne were observed to be 9.8%, 15.2%, and 2.2% among patients assigned to receive upadacitinib at doses of 15 mg, 30 mg, and placebo, respectively (Mendes-Bastos et al., 2022). In contrast, upadacitinib can reduce the risk of atopic dermatitis, and the higher the dose, the lower the incidence of atopic dermatitis (RR = 2.16; 95% CI, 1.15–4.04; p = 0.02), which is consistent with the effectiveness of upadacitinib in patients with atopic dermatitis. The results of a study demonstrated that upadacitinib treatment in patients with moderate-to-severe juvenile atopic dermatitis exhibited a prolonged and sustained efficacy, lasting for up to 76 weeks (equivalent to 17.5 months) (Paller et al., 2024).

There was no statistically significant difference in the incidence of NMSC between the two doses of upadacitinib and placebo. In addition, one study has shown that the combination therapy of JAK inhibitors and methotrexate (MTX) does not increase the risk of malignant tumors in RA patients compared with the use of MTX alone, indicating that this combination therapy has overall acceptable safety (Solipuram et al., 2021). However, the results of this study revealed a significantly higher incidence of malignancies other than NMSC with the 30 mg dose compared to placebo (RR = 3.42; 95% CI, 1.20–9.75; p = 0.02). Nonetheless, malignancies other than NMSC occurred only in 0.59% of cases at the 30 mg dose, with twelve reported cases among 2043 participants. In a study examining the long-term safety of upadacitinib, only one case of stage IVa squamous carcinoma of the tongue (malignancies other than NMSC) was observed in a patient with ankylosing spondylitis; however, it is worth noting that this particular patient had a history of smoking and had been exposed to upadacitinib for less than 5 months (Burmester et al., 2023). Therefore, the sample size of low-incidence events such as malignancies other than NMSC is limited, which may affect the statistical power. The extrapolation of the results requires caution and further exploration is still needed.

The findings of this study demonstrate that neither 15 mg nor 30 mg of upadacitinib treatment pose any risk of MACE or VTE. The results of the multinational, multicenter safety study on upadacitinib for ulcerative colitis treatment revealed that both the upadacitinib 30 mg group and placebo group had one confirmed MACE (<1% of patients), while both the 15 mg and 30 mg groups experienced two VTEs (1% of patients) (Vermeire et al., 2023). It is noteworthy that all identified MACE and VTE occurred in patients with known risk factors. It cannot be ignored that MACE or VTE is a rare adverse reaction. Coupled with the short follow-up time, the statistical power is limited. Therefore, long-term monitoring data are still needed for a comprehensive assessment.

As a second-generation JAK inhibitor, the safety profile of upadacitinib has garnered significant attention. The black box warning imposed on it has generally restricted its clinical application. Given the broad spectrum of indications for JAK inhibitors and the substantial patient population involved, the FDA’s cautious approach is understandable. Existing research data support the performance of upadacitinib in terms of safety, and the results of this study also provide evidence for this. With the accumulation of more research evidence in the future, it will help to further assess whether adjustments are needed to the current black box warning content.

However, several limitations in our study warrant further improvement. Firstly, our analysis was based on study-level data, which precluded a comprehensive assessment and inclusion of individual patient-level confounding factors. Secondly, the follow-up period for the included studies spanned only tens of weeks, whereas safety outcomes typically require long-term data for confirmation. Thirdly, this study encompassed patients with diverse underlying conditions, some results were subgroup analyzed by disease type, but the potential influence of these conditions on the results was not fully elucidated.

## Conclusion

This systematic review and meta-analysis elucidates the incidence and risk of adverse events associated with upadacitinib across diverse patient cohorts enrolled in RCTs, while also highlighting the disparity in adverse event risks between different dosages of upadacitinib. The results demonstrated a generally favorable safety profile in patients treated with either 15 mg or 30 mg doses of upadacitinib. Upadacitinib treatment was associated with an increased risk of hepatic disorder, neutropenia, acne, herpes zoster, and increased CPK levels. Notably, the risks of hepatic disorder, neutropenia, and acne also exhibited a dose-dependent relationship. However, there was no significant association between upadacitinib treatment and an elevated risk of renal dysfunction, NMSC, MACE, or VTE. These findings may serve as an evidence base for potential future modifications or removal of the FDA’s black box warning for upadacitinib.

## Data Availability

The original contributions presented in the study are included in the article/Supplementary Material, further inquiries can be directed to the corresponding author.
